# A novel variant in the *ROR2* gene underlying brachydactyly type B: a case report

**DOI:** 10.1186/s12887-022-03564-z

**Published:** 2022-09-05

**Authors:** Jiaqi Shao, Yue Liu, Shuyang Zhao, Weisheng Sun, Jie Zhan, Lihua Cao

**Affiliations:** 1 College of Kinesiology, Shenyang Sport University, No. 36 Jinqiansong East Road, Sujiatun District, Shenyang, 110102 China; 2 Hand SurgeryCentral Hospital Affiliated to Shenyang Medical CollegeTiexi District, Dept.4No. 5 Nanqi West Road, Shenyang, 110024 China

**Keywords:** Brachydactyly type B1, *ROR2*, Whole-exome sequencing, Variant

## Abstract

**Background:**

Brachydactyly type B is an autosomal dominant disorder that is characterized by hypoplasia of the distal phalanges and nails and can be divided into brachydactyly type B1 (BDB1) and brachydactyly type B2 (BDB2). BDB1 is the most severe form of brachydactyly and is caused by truncating variants in the receptor tyrosine kinase–like orphan receptor 2 (*ROR2*) gene.

**Case presentation:**

Here, we report a five-generation Chinese family with brachydactyly with or without syndactyly. The proband and her mother underwent digital separation in syndactyly, and the genetic analyses of the proband and her parents were provided. The novel heterozygous frameshift variant c.1320dupG, p.(Arg441Alafs*18) in the *ROR2* gene was identified in the affected individuals by whole-exome sequencing and Sanger sequencing. The c.1320dupG variant in *ROR2* is predicted to produce a truncated protein that lacks tyrosine kinase and serine/threonine- and proline-rich structures and remarkably alters the tertiary structures of the mutant ROR2 protein.

**Conclusion:**

The c.1320dupG, p.(Arg441Alafs*18) variant in the *ROR2* gene has not been reported in any databases thus far and therefore is novel. Our study extends the gene variant spectrum of brachydactyly and may provide information for the genetic counselling of family members.

**Supplementary Information:**

The online version contains supplementary material available at 10.1186/s12887-022-03564-z.

## Background

Brachydactyly (BD) is an autosomal dominant hand and foot malformation that is characterized by abnormally shortened or missing phalanges and/or metacarpals/metatarsals [1]. It may occur as an isolated trait or as part of a complex malformation syndrome [2]. According to the Bell classification, heritable isolated BDs have been classified into subtypes A to E according to their patterns of skeletal involvement [3]. Clinical features and pathogenic genes of isolated BDs are listed in Table 1 [4–20]. Brachydactyly type B1 (BDB1, OMIM 113,000) is the most severe form of brachydactyly and is characterized by shortening or hypoplasia of the distal and middle phalanges of digits 2 through 5 with or without nail dysplasia, fusion of the middle and distal phalanges, variable degrees of distal and proximal symphalangism, and a broad or bifid thumb. The feet are similarly but less severely affected [13–15].

**Table 1:** Types of isolated brachydactyly

Phenotype		Phenotype MIM number	Clinical features	Pathogenic genes	Gene/Locus MIM number	Reference
BDA1	BDA1	112,500	Short middle phalanges of all fingers and short proximal phalanx of the thumb. The middle phalanx may be absent in severe cases	*IHH*	600,726	[4]
	BDA1B	607,004		5p13.3-p13.2	None	[5]
	BDA1C	615,072		*GDF5*	601,146	[6]
	BDA1D	616,849		*BMPR1B*	603,248	[7]
BDA2		112,600	Short middle phalanx with clinodactyly of the index fingers and second toes	*BMPR1B*	603,248	[8]
				*GDF5*	601,146	[9]
				*BMP2*	112,261	[10]
BDA3		112,700	Short middle phalanx of the little fingers with or without clinodactyly	*HOXD13*	142,989	[11]
BDA4		112,800	Short middle phalanx of the index and little fingers. The middle phalanges of the lateral four toes are frequently involved	*HOXD13*	142,989	[12]
BDB1		113,000	Hypoplastic or absent distal phalanges of digits 2–5 with or without nail dysplasia, the fusion of the middle and distal phalanges, abroad or bifid thumb, and sometimes distal and proximal symphalangism or syndactyly	*ROR2*	602,337	[13–15]
BDB2		611,377	Hypoplasia of distal phalanges with distal and proximal symphalangism, fusion of carpal or tarsal bones, and partial cutaneous syndactyly	*NOG*	602,991	[16]
BDC		113,100	Brachymesophalangy of the index, middle and little fingers with preservation of the ring finger. The index and middle fingers show hyperphalangism and their most proximal phalanges have abnormal configuration lending to ulnar deviation. The thumb metacarpals are slightly short	*GDF5*	601,146	[17]
BDD		113,200	Stub thumbs (short distal phalanges of the thumbs). The big toes may be similarly affected	*HOXD13*	142,989	[18]
BDE1		113,300	Short metacarpal IV, with/without short metatarsal IV (possible involvement of an isolated metatarsal)	*HOXD13*	142,989	[18]
BDE2		613,382	Short metacarpals IV and V (and metatarsals) with short distal phalanx of the thumb	*PTHLH*	168,470	[19]
BDE3		None	Short metacarpals without phalangeal involvement	None	None	[20]

BDB1 is usually caused by a heterozygous variant in the receptor tyrosine kinase (RTK)-like orphan receptor 2 (*ROR2*) gene (OMIM 602,337), which is located on chromosome 9q22.31 [14, 21]. The *ROR2* gene contains nine exons and spans a genomic length of approximately 228 kb. The ROR2 protein contains 943 amino acids and belongs to the ROR family of RTKs. It consists of an extracellular region, a transmembrane section, and an intracellular region [22]. Heterozygous variants that truncate the intracellular portion of ROR2, either the N-terminal or C-terminal of the TK domain, are the leading causes of BDB1 [22, 23]. In addition, homozygous or compound heterozygous variants in the *ROR2* gene are responsible for recessive Robinow syndrome [22].

In recent years, whole-exome sequencing has become a routine strategy for discovering potential causal variants in inherited Mendelian disorders. It provides a cost-effective, fast-track approach to variant discovery and considerably increases the overall diagnostic rate. In this study, we identified the disease-causing variant in a five-generation Chinese family with BDB1 using whole-exome sequencing and Sanger sequencing. Pathogenicity was inferred by bioinformatic analysis.

## Case presentation

**Fig. 1:**
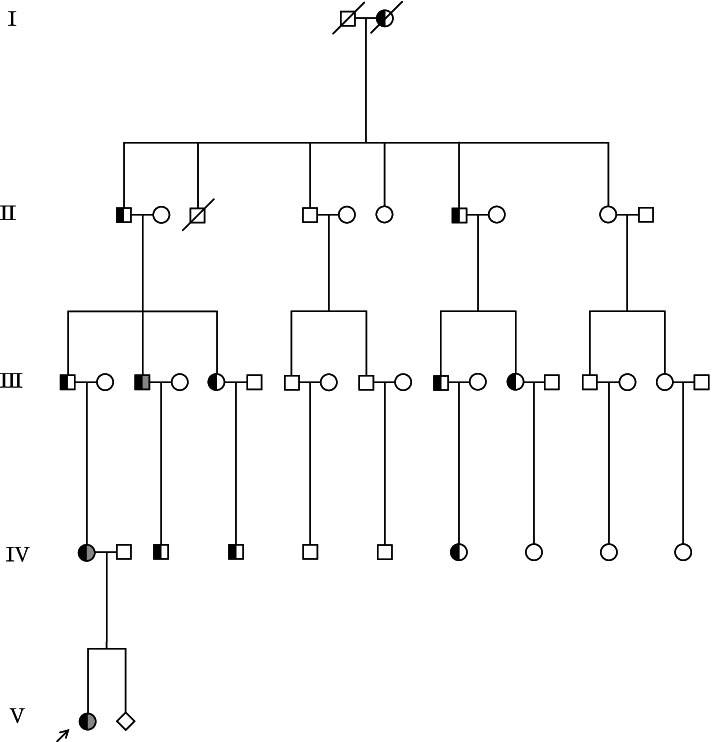
Pedigree of the family with BDB1. Black shading on the left half represents brachydactyly, and grey shading on the right half represents syndactyly. The proband is marked by an arrow

**Fig. 2:**
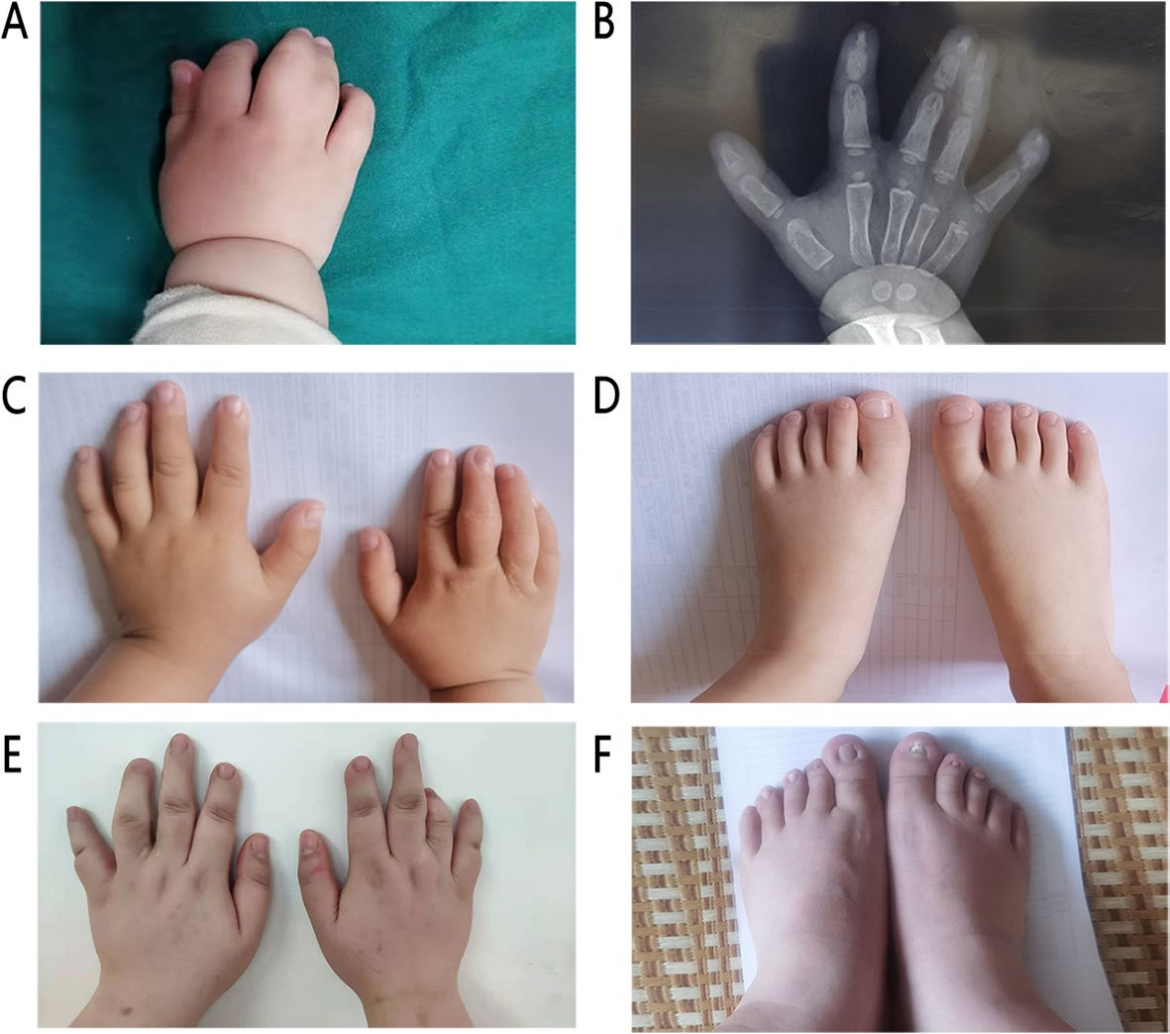
Phenotype of the proband and her mother. **a** A photograph of the proband's right hand before surgery. **b** An X-ray of the proband’s right hand before surgery. **c** A picture of the proband’s hands after the operation. **d** A photograph of the proband's feet. **e** A photograph of the mother’s hands after the operation. **f** A photograph of the mother’s feet

A five-generation Chinese family with BDB1 was recruited at Central Hospital Affiliated to Shenyang Medical College. Clinical information of all family members and blood specimens from the proband and her parents were obtained. Among the 13 affected individuals, 10 had brachydactyly, and the remaining three had brachydactyly with syndactyly (Fig. 1). The 1-year-old female proband was physically examined and received a radiographic examination of the right hand before surgery. She exhibited bilateral shortening and hypoplasia of the distal and middle phalanges of digits 2 to 5 with cutaneous syndactyly of right fingers 3 to 4. The proband underwent digital separation on her right hand. Her mother underwent digital separation on both hands before enrolment in this study. Therefore, her phenotypic information was determined after reviewing her medical records and performing a physical examination. She had shortening/hypoplasia of the distal and middle phalanges of digits 2 to 5 with cutaneous syndactyly of fingers 3 to 4 on both hands, and she also had a broad hallux on both feet with nail dysplasia (Fig. 2). The phenotypic information of the other family members was provided by the proband’s mother.

**Fig. 3:**
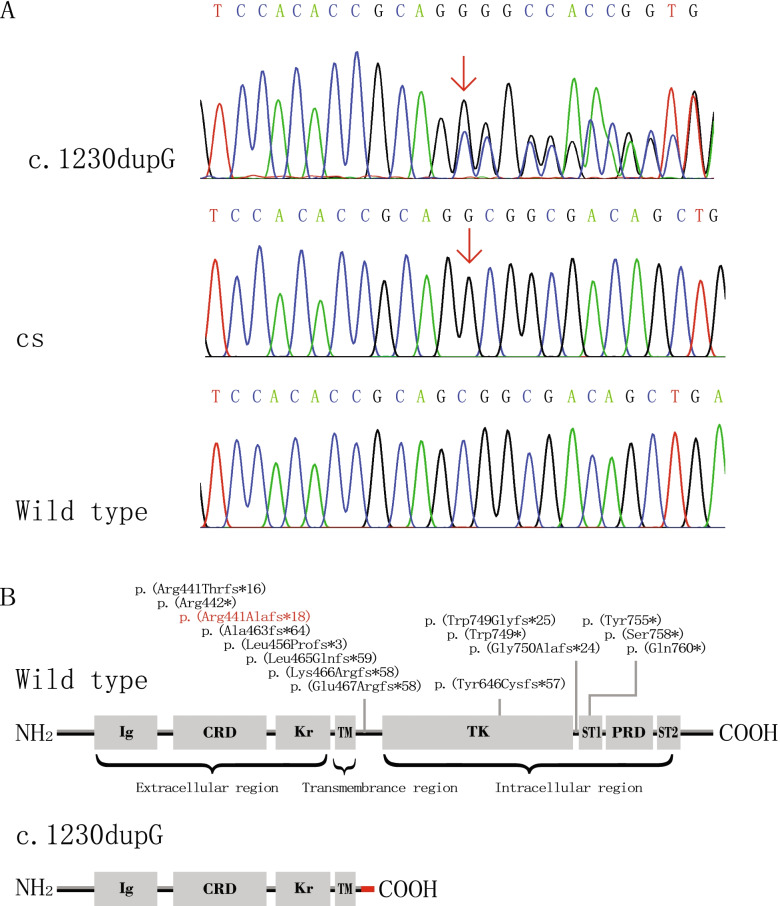
Gene variant analysis of a family with BDB1. **a** The novel heterozygous variant c.1320dupG, p.(Arg441Alafs*18) in *ROR2* was verified by Sanger sequencing. The variant is marked by a red arrow. CS: Clone sequencing. **b** A schematic diagram showing the encoded domain structure of the *ROR2* gene. A recurrent variant is marked in black, and the novel variant identified in this study is highlighted in red. This *ROR2* variant results in the loss of the whole intracellular region

**Fig. 4:**
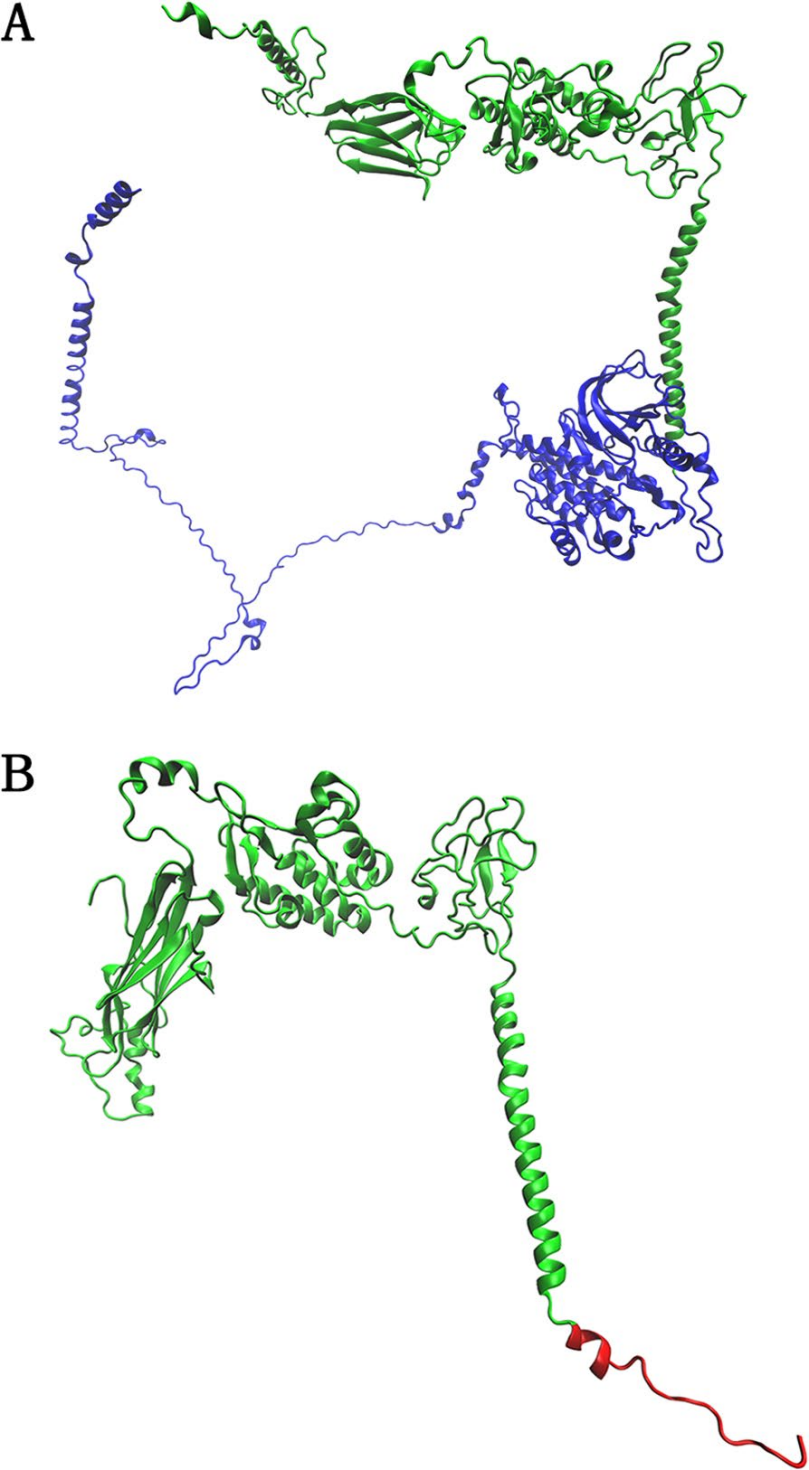
Three-dimensional structures of the wild-type and variant ROR2 proteins. Molecular modelling by Robetta showing that p.(Arg441Alafs*18) remarkably altered the tertiary structures of the remaining peptide chain of the ROR2 protein. **a** Wild-type; **b** Mutant type

Genomic DNA was extracted from the peripheral venous blood of the proband using the QIAamp DNA Blood Mini Kit (QIAGEN, Hilden, Germany) according to the manufacturer’s protocol and the AI Exome V1 Kit (iGeneTech, AIEV1). The extracted DNA was used for whole-exome capture and enrichment. Exome sequencing was performed on the NovaSeq 6000 sequencing platform (Illumina). Gene variants were initially annotated using the wANNOVAR tool (http://wannovar.wglab.org/). Those variants with minor allele frequencies greater than 0.01 according to public genomic databases, such as 1000Genomes, dbSNP, Exome Variant Server, gnomAD, and the Exome Aggregation Consortium were filtered out. The c.1320dupG, p.(Arg441Alafs*18) variant was identified in exon 8 of *ROR2* by whole-exome sequencing and Sanger sequencing. This variant results in a frameshift at amino acid position 441. This frameshift creates a polypeptide chain of 17 new amino acids and a premature termination codon at amino acid position 458. To read the duplication sequencing chromatogram more clearly, the purified PCR fragment of the proband was inserted into the pMD-18 T vector. The selected clones were sequenced using the universal primers M13F and M13R (Fig. 3a). At the protein level, the c.1320dupG variant is predicted to produce a truncated protein that lacks tyrosine kinase and serine/threonine- and proline-rich structures, resulting in a loss of the whole intracellular region (Fig. 3b). Three-dimensional structures of wild-type and mutant ROR2 proteins were generated by the Robetta online server (http://robetta.org/) and validated using different parameters, such as the Ramachandran plot, ERRAT score, confidence and *P *value [24, 25]. The first two parameters were calculated using Structure Analysis and Verification Server (SAVES) version 6.0 (https://saves.mbi.ucla.edu/), and the last two parameters were determined using the ModFOLD Model Quality Assessment Server (https://www.reading.ac.uk/bioinf/ModFOLD/) [26–29]. Illustrations were prepared using VMD (www.ks.uiuc.edu/Research/vmd/). Molecular modelling shows that p.(Arg441Alafs*18) severely affects the tertiary structure of the remaining peptide chain of ROR2 (Fig. 4). The Ramachandran plot shows that the total number of residues in the most favoured regions for the wild-type and mutant ROR2 proteins was 688 (85.9%) and 330 (86.2%), respectively, and the corresponding number of residues in disallowed regions was 5 (0.6%) and 4 (1.0%), respectively. The ERRAT scores of wild-type and mutant ROR2 proteins were 89.732 and 86.9863, respectively. The confidence scores and *P* values of the wild-type (0.04122) and mutant proteins (0.05723) were less than 0.1 (Additional file 1). The above results indicate that the three-dimensional structures of the wild-type and mutant ROR2 proteins we predicted were reliable.

## Discussion and conclusion

ROR2 is a single-pass transmembrane protein. The domain structure of ROR2 is composed of an extracellular immunoglobulin-like domain (Ig, aa residues 55–145), frizzled-like cysteine-rich domain (FRZ or CRD, aa residues 169–303), Kringle domain (Kr, aa residues 316–394), transmembrane domain (TM, aa residues 404–424), and intracellular tyrosine kinase (TK, aa residues 473–746), and serine/threonine-rich (ST1, aa residues 753–782; ST2, aa residues 859–882) and proline-rich (PRD, aa residues 784–857) structures [22]. The extracellular region interacts with Wnt5a, which binds to the CRD region of ROR2 [30]. Both canonical and noncanonical Wnt5a/Ror2 signalling play an important role in limb skeletal development and morphogenesis [30–32]. The intracellular region of ROR2 is associated with many factors, such as 14–3-3 protein beta/alpha, nuclear receptor subfamily 2 group C member 2, proto-oncogene tyrosine-protein kinase Src, Wilms tumour protein 1-interacting protein, and SHC-transforming protein 1 [23, 30, 33–36]. Activation of ROR2 kinase requires the intracellular C-terminal region for recruitment of the nonreceptor kinase Src [34, 37].

Up to the time of this article’s publication, a total of 15 different variants in the *ROR2* gene were found to be associated with BDB1 (Table 2). All the documented BDB1-causing *ROR2* variants were nonsense or frameshift variants and were clustered in the last two exons (exons 8 and 9) or in the last intron (intron 8). Thus, the mutant mRNAs were able to escape degradation by nonsense-mediated decay, resulting in a partial or complete truncation of the intracellular portion of the ROR2 protein [38]. These mutations are believed to have a specific gain-of-function effect, not a simple haploinsufficiency [39]. The BDB1-related variants can be divided into distal and proximal variants. The distal variant that is located after TK produces a truncated protein that lacks the ST1, ST2, and PRD domains, leading to a more severe phenotype [14, 40]. In contrast, the proximal variant results in a loss of the whole intracellular region, causing a less severe but more variable phenotype [37].

**Table 2:** Clinical manifestation of *ROR2* causing BDB1

Number	Nucleotide change	Predicted amino acid change	Clinical features	Reference
1	c.1324C > T	p.(Arg442*)	Homozygous individuals exhibited features of recessive Robinow syndrome in conjunction with severe recessive brachydactyly	[39]
2	c.2246G > A	p.(Trp749*)	The patients have classical BDB1 with bilateral symmetry of the deformities	[41]
3	c.2247G > A	p.(Trp749*)	An English family with typical BDB1. In addition, they have a short philtrum and a prominent nose with a high bridge and bulbous tip; A Chinese family had classical BDB1	[42, 43]
4	c.2265C > A	p.(Tyr755*)	These families displayed classical BDB1, with (or without) a prominent nose with a bulbous tip, wide-spaced eyes, and a short philtrum	[13, 40, 41, 44]
5	c.2273C > A	p.(Ser758*)	Classical BDB1	[37]
6	c.2278C > T	p.(Gln760*)	The proband lacked distal phalanges and nails and had hypoplastic middle phalanges of digits 2–5	[14]
7	c.1321_1325delCGGCG	p.(Arg441Thrfs*16)	Heterozygous individuals exhibited classical BDB1, whereas homozygous individuals showed severe skeletal defects, primarily affecting the distal limbs and the spine	[14]
8	c.1394_1395delTC	p.(Leu465Glnfs*59)	No detailed clinical description	[45]
9	c.1397_1398delAA	p.(Lys466Argfs*58)	Variable degrees of hypoplastic or shortened distal phalanges on digits 2–5 of the hands. Very few of the toes were affected	[46]
10	c.1937_1943delACAAGCT	p.(Tyr646Cysfs*57)	Homozygous individuals exhibited features of recessive Robinow syndrome in conjunction with severe brachydactyly	[47]
11	c.2244delC	p.(Trp749Glyfs*25)	The patients exhibited atypical BDB1 and cutaneous syndactyly of varying degrees	[38]
12	c.2249delG	p.(Gly750Alafs*24)	The morphologic abnormalities were more severe, such as a bifid thumb and syndactyly of the central digits (digits 2 and 4)	[41]
13	c.1366dupC	p.(Leu456Profs*3)	The patients exhibited bilateral or unilateral fourth finger distal symphalangism with (or without) absence of the distal phalanx or fusion of the phalangeal bones on the hand radiograph	[48]
14	c.1398dupA	p.(Glu467Argfs*58)	They exhibited the absence of distal phalanges of the 4^th^ finger and hypoplasia of distal phalanges of fingers 2, 3, and 5, with or without nail dysplasia	[14]
15	c.1386 + 3_1386 + 5delCTCins19	p.(Ala463fs*64)	No detailed clinical description	[14]

In this study, the novel proximal frameshift variant c.1320dupG, p.(Arg441Alafs*18) in the *ROR2* gene was identified in a Chinese family with BDB1. This variant is predicted to produce a premature termination codon at amino acid position 458 and a new polypeptide consisting of 17 amino acids at the variant position. The location of this variant was very similar to the locations of c.1321_1325delCGGCG, p.(Arg441Thrfs*16), and c.1324C > T, p.(Arg442*), but their phenotypes were different. Individuals heterozygous for c.1321_1325delCGGCG, p.(Arg441Thrfs*16) exhibited bilateral hypoplasia of the distal and middle phalanges of the fingers and toes 2 to 5 to varying degrees, with or without hypoplasia of the nails. In contrast, individuals homozygous for c.1321_1325delCGGCG, p.(Arg441Thrfs*16), showed severe skeletal defects that primarily affected the distal limbs and the spine [14]. Interestingly, a heterozygous c.1324C > T, p.(R442*) carrier had a normal limb phenotype, and a homozygous c.1324C > T, p.(R442*) individual exhibited features of recessive Robinow syndrome in conjunction with severe recessive brachydactyly [39]. The c.1320dupG, p.(Arg441Alafs*18) variant in the *ROR2* gene reported by our study caused bilateral or unilateral shortening/hypoplasia of the distal and middle phalanges of digits 2 to 5 with or without cutaneous syndactyly of fingers 3 to 4. The relationship between the *ROR2* gene variants and phenotypes is not completely clear. Previous studies suggest that BDB1 variants result in a gain in function, whereas Robinow syndrome variants result in a loss of function [14, 49]. Further research should investigate the reasons for the different phenotypes resulting from these similar gene variants.

In conclusion, we report the novel variant c.1320dupG, p.(Arg441Alafs*18) in the *ROR2* gene in a Chinese family with BDB1. Our study extends the gene variant spectrum of BDB1 and provides information for the genetic counselling of family members.

## Supplementary Information

**Additional file 1. **The results of three-dimensional structures of wild-type and mutant ROR2 proteins were validated using different parameters.

## Data Availability

The datasets generated and/or analysed during the current study are available in the NCBI Sequence Read Archive (SRA) repository (accession number: SRP390837).

## Abbreviations

BD: Brachydactyly
BDB1: Brachydactyly type B1
RTK: Receptor tyrosine kinase
Ig: Immunoglobulin-like domain
FRZ or CRD: Frizzled-like cysteine-rich domain
Kr: Kringle domain
TM: Transmembrane domain
TK: Intracellular tyrosine kinase
ST1 ST2: Serine/threonine-rich
PRD: Proline-rich
