# Supplementary material for: A novel variant in the ROR2 gene underlying brachydactyly type B: a case report
Source: BMC Pediatr. 2022 Sep 5;22:528. doi: 10.1186/s12887-022-03564-z (PMC9446770; doi:10.1186/s12887-022-03564-z)

The results of three-dimensional structures of wild-type and mutant ROR2 proteins were validated using different parameters.

1. Ramachandran plot

(1) Ramachandran plot-Wild type


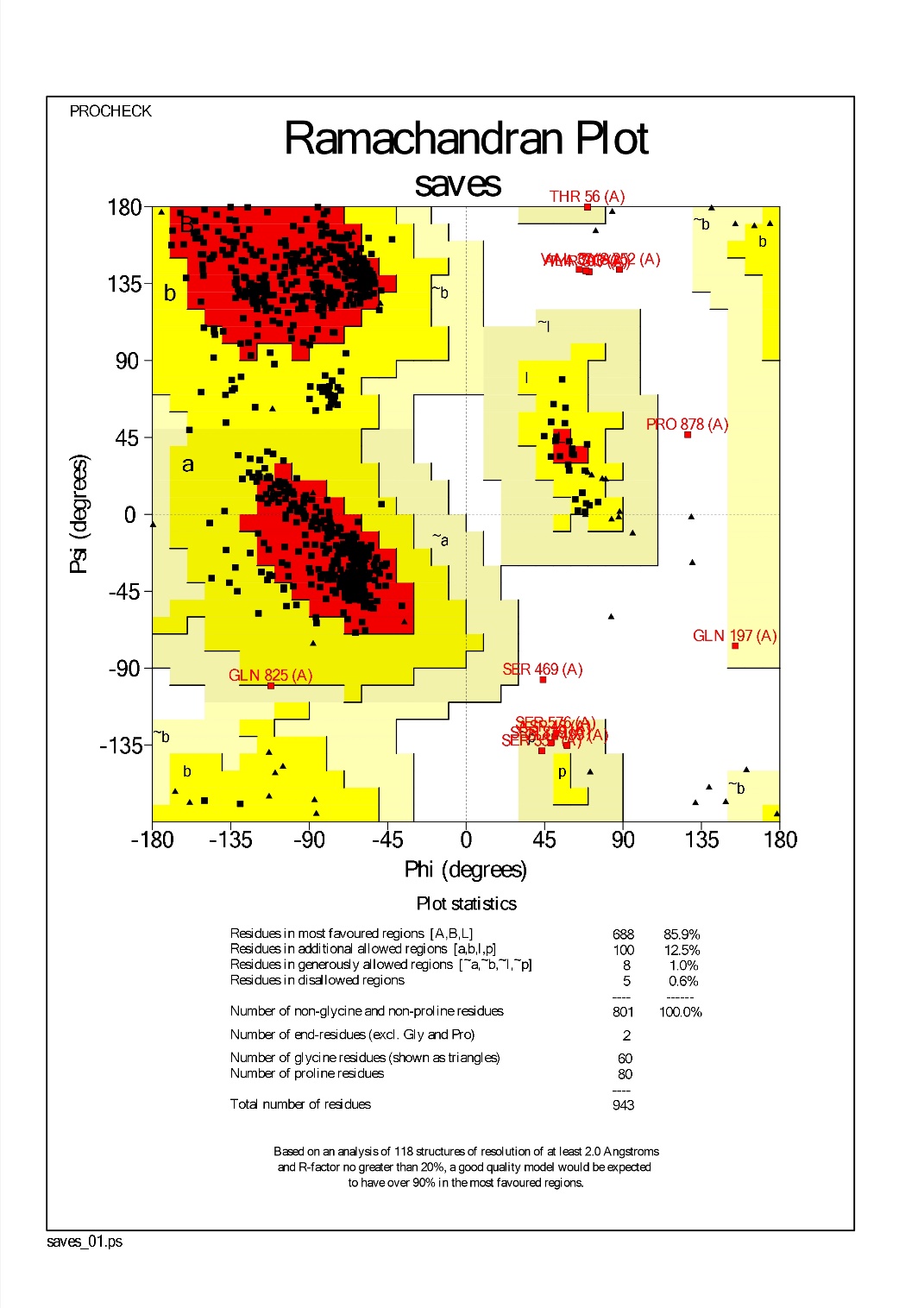


(2) Ramachandran plot-p.(Arg441Alafs*18)


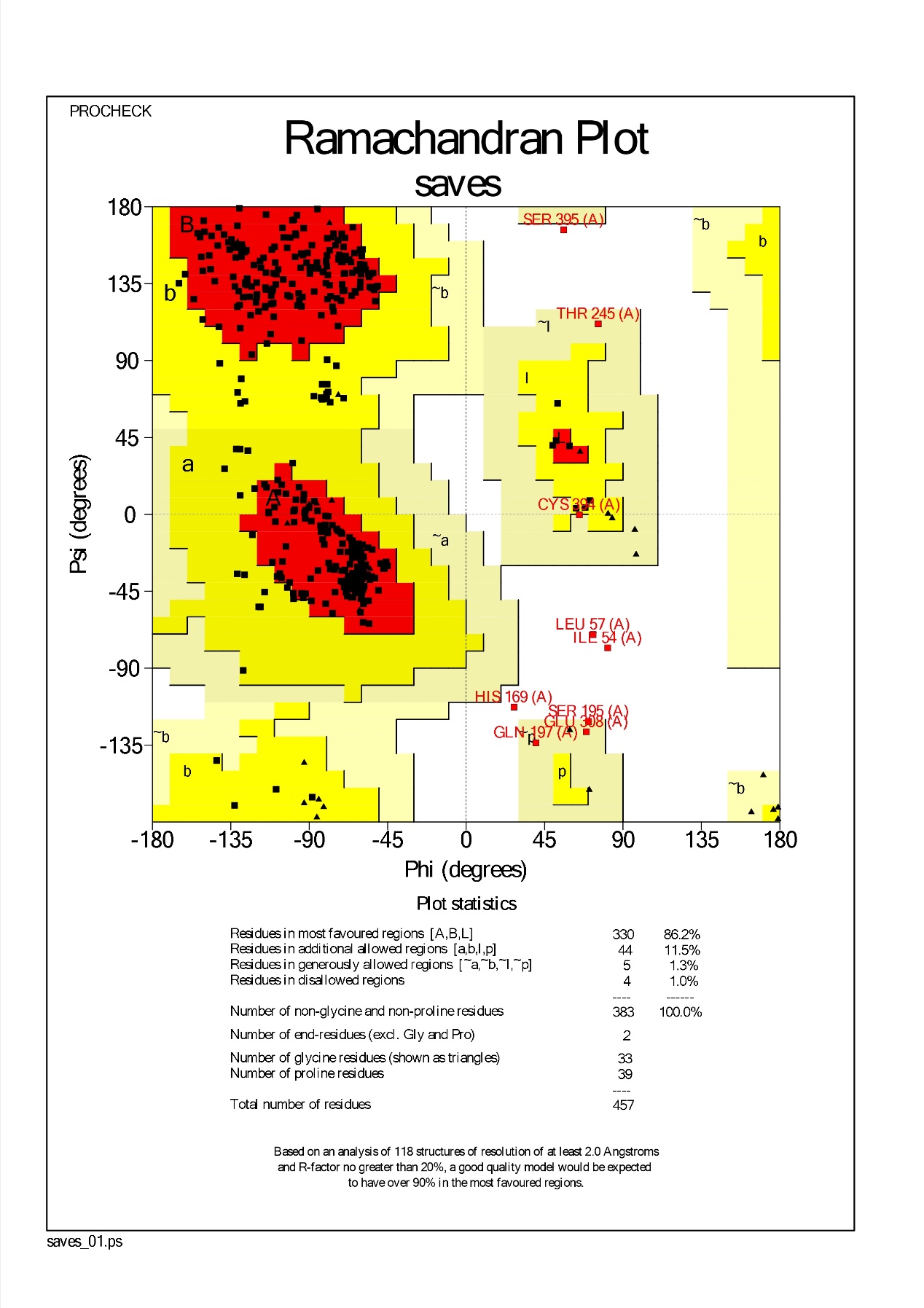


2. ERRAT score

(1) ERRAT score-Wild type


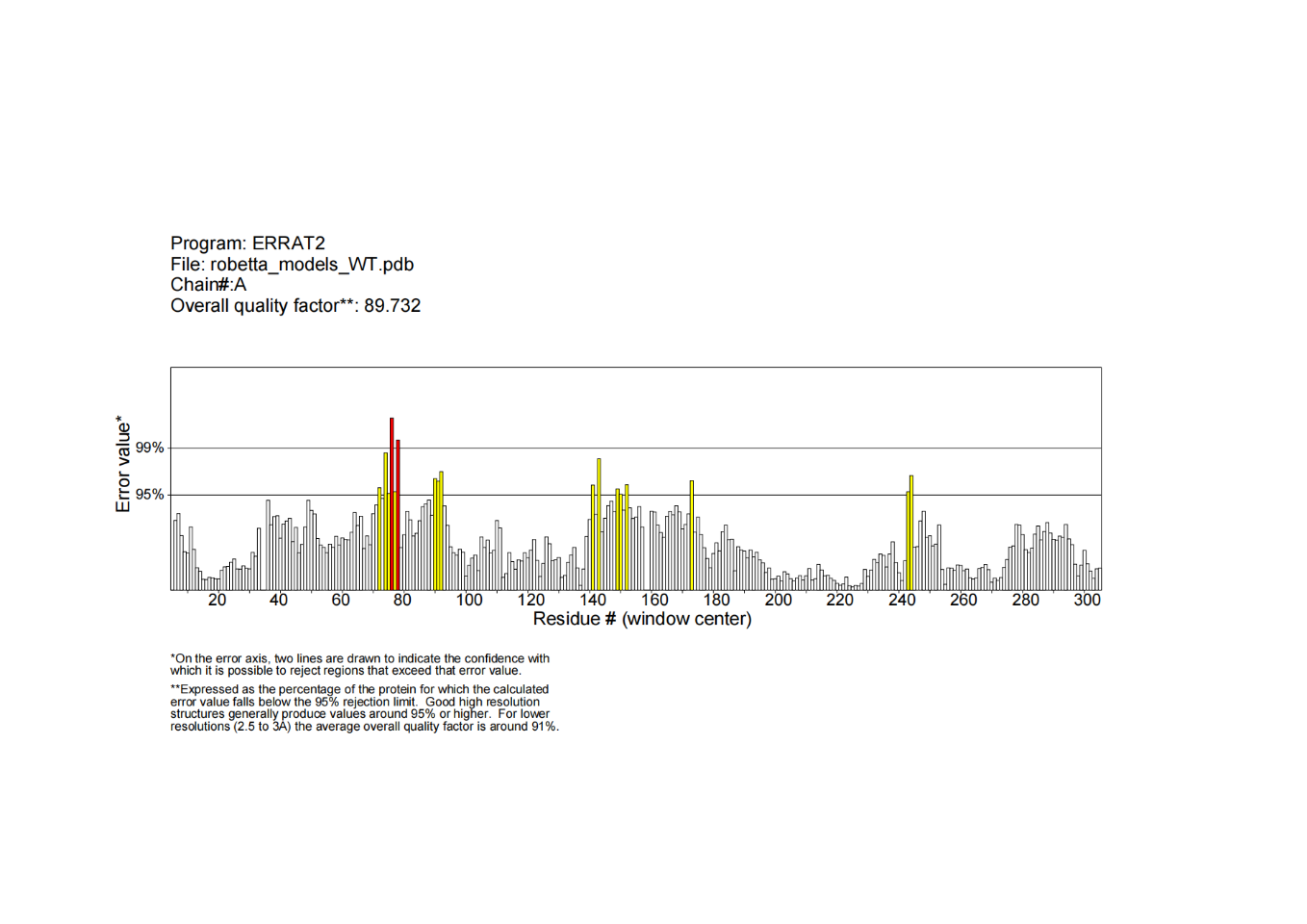


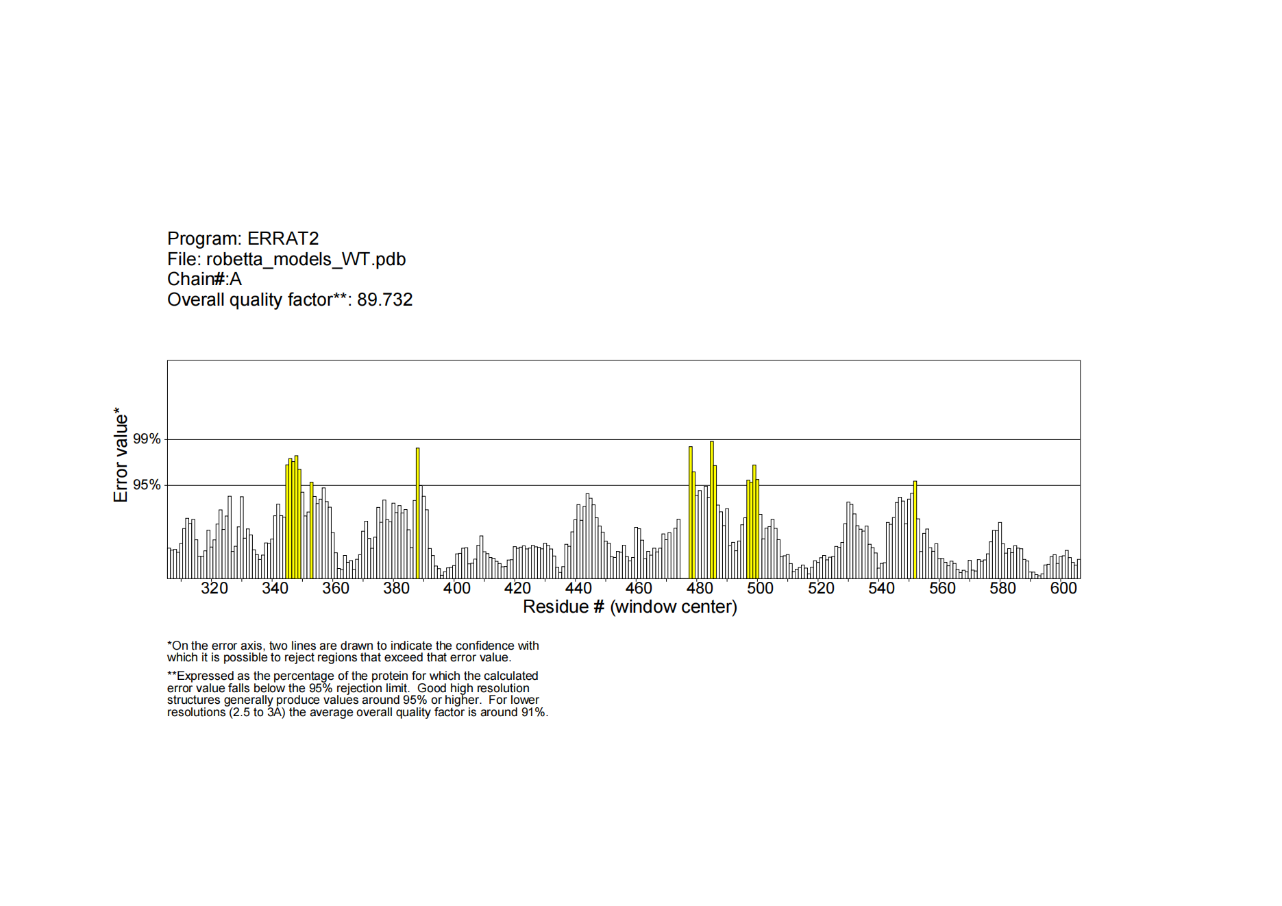


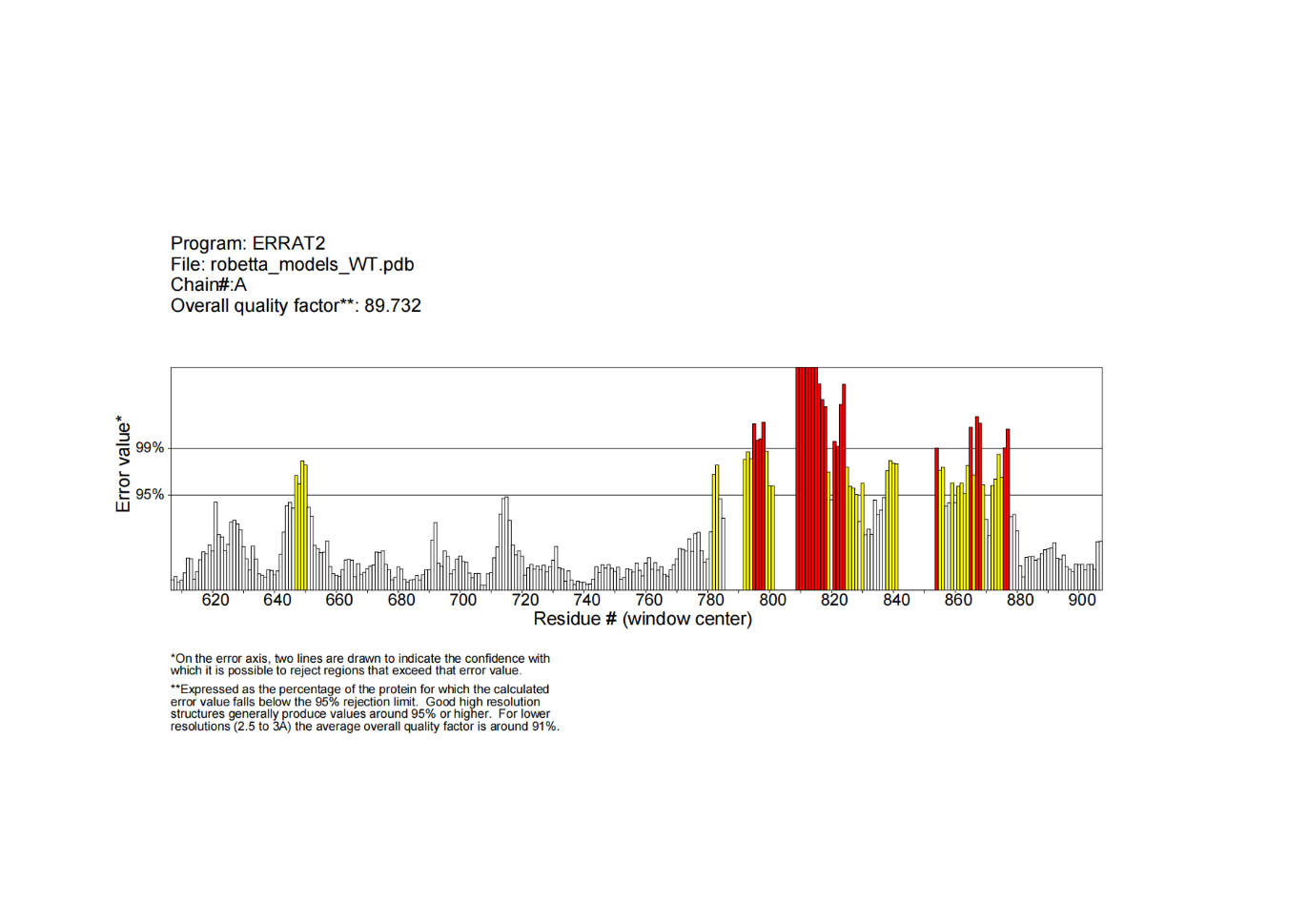


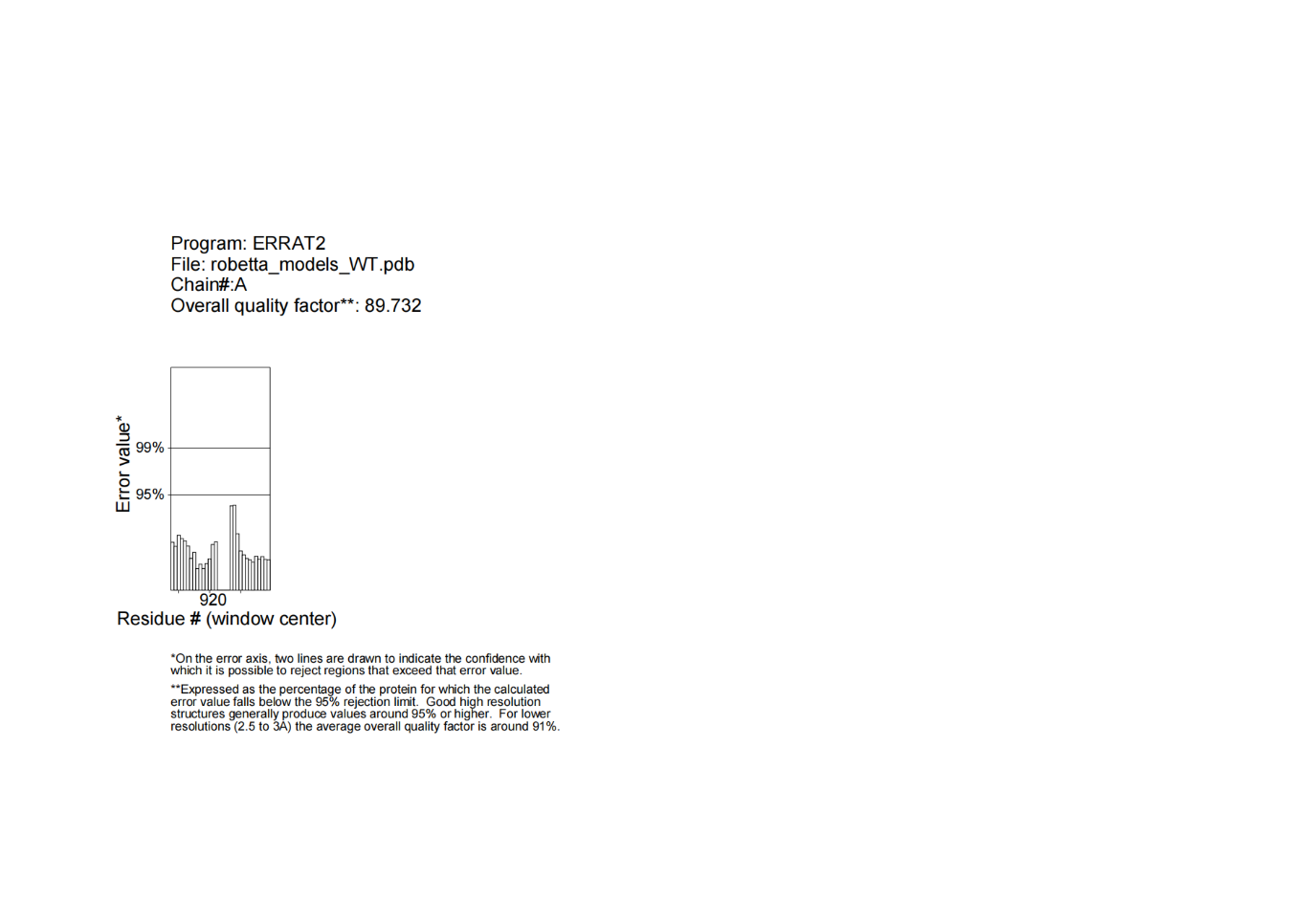


(2) ERRAT score-p.(Arg441Alafs*18)


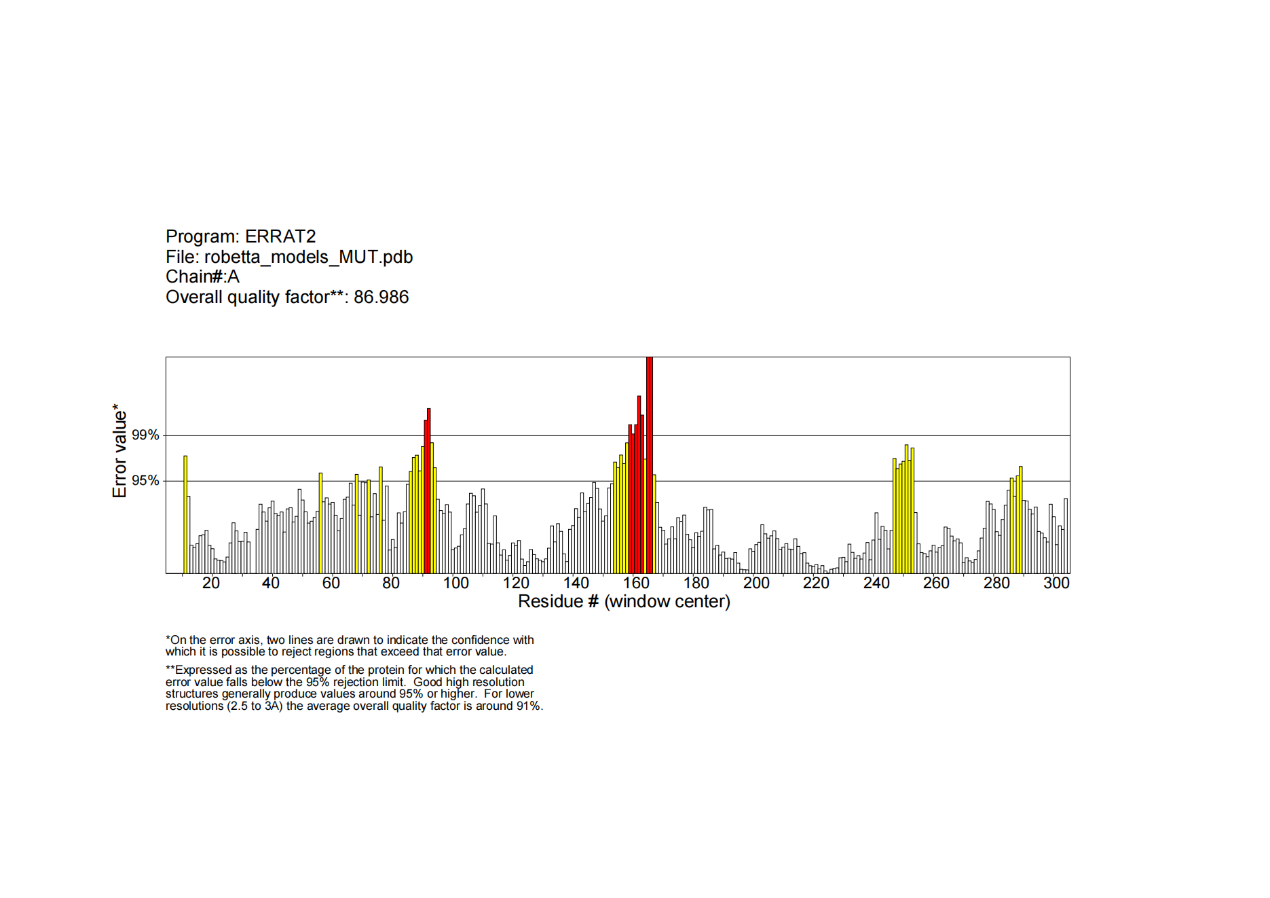


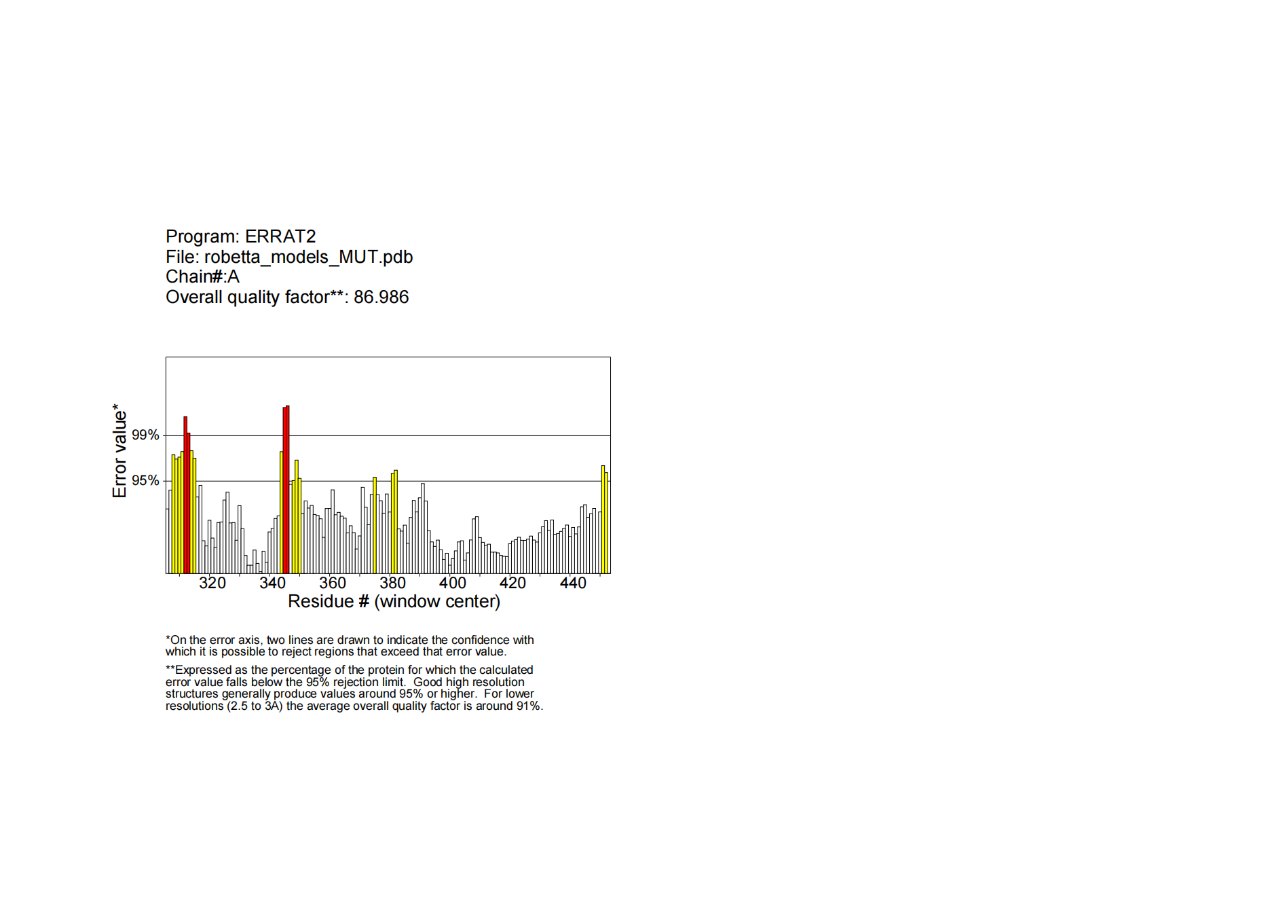


3. Confidence and *P*-value

(1) Confidence and *P*-value-Wild type


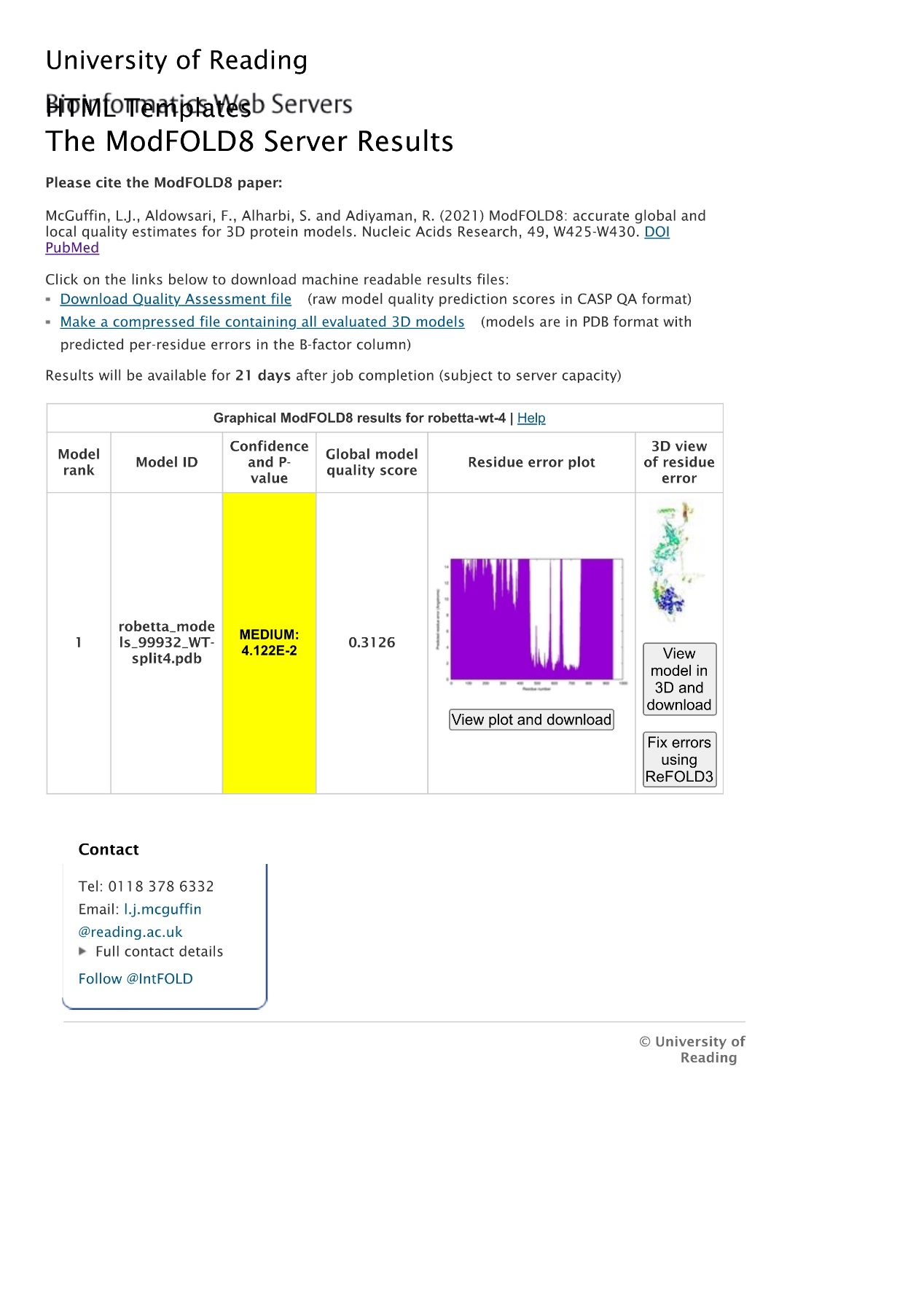


(2) Confidence and *P*-value-p.(Arg441Alafs*18)


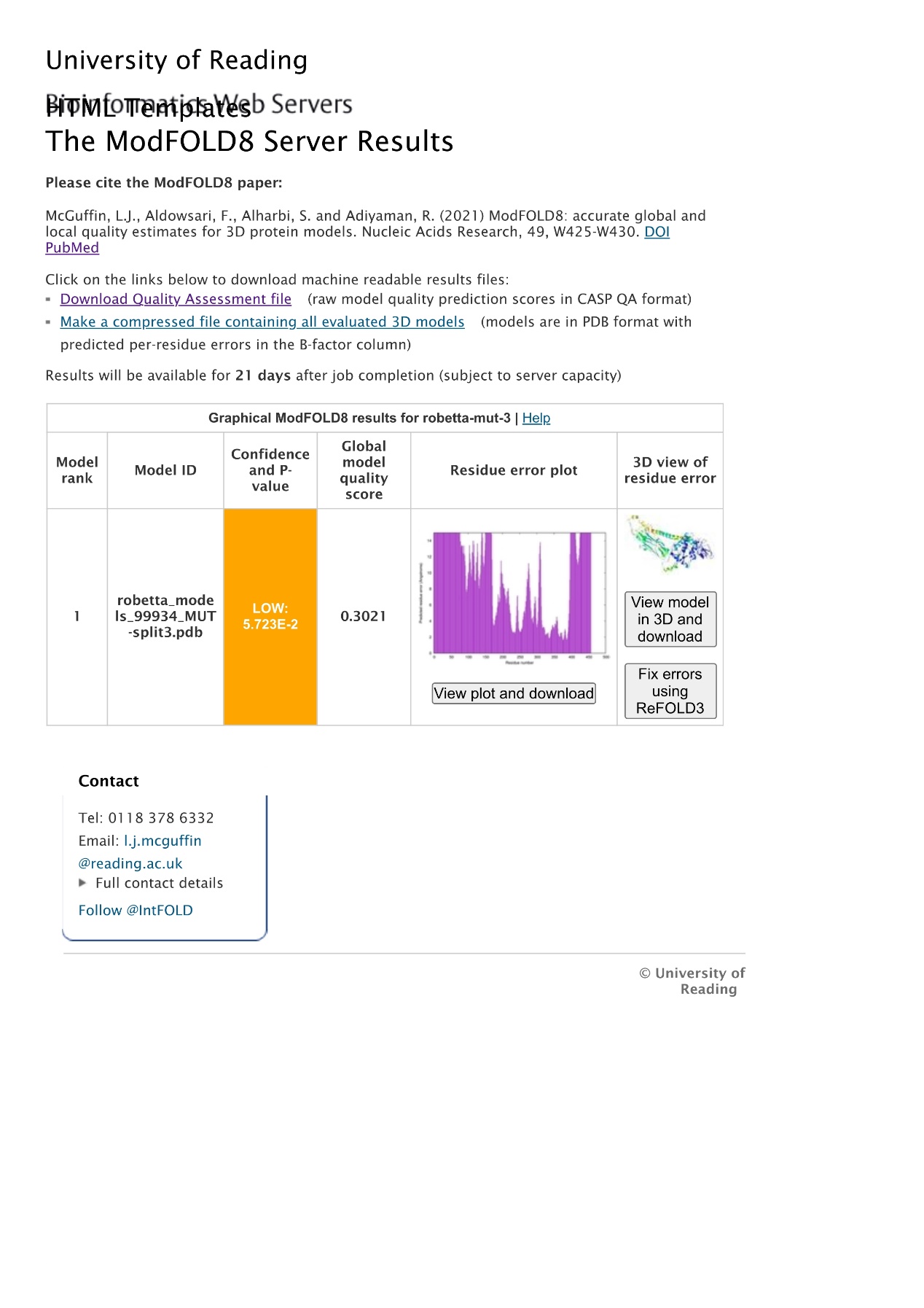

Supplement: Supplementary file 1 — Additional file 1. The results of three-dimensional structures of wild-type and mutant ROR2 proteins were validated using different parameters. [file 12887_2022_3564_MOESM1_ESM.docx]
